# Neuraxial analgesia interfered with the circadian rhythm of labor: a propensity score matched cohort study

**DOI:** 10.1186/s12884-021-04311-5

**Published:** 2022-01-03

**Authors:** Li Wang, Xuyuan Ma, Le Chen, Fangfang Jiang, Jie Zhou

**Affiliations:** 1 Department of Obstetrics and Gynecology, Perinatal Medical Center, The Fifth Affiliated Hospital of Sun Yat-sen University, Zhuhai, 519000 China; 2 Department of Anesthesiology, Perioperative and Pain Medicine, Brigham and Women’s Hospital, Harvard Medical School, 75 Francis St, Boston, MA 02115 USA

**Keywords:** Neuraxial analgesia, Labor, Circadian rhythm, Induction, Oxytocin

## Abstract

**Objectives:**

To investigate whether neuraxial analgesia and other medical interventions have effects on the circadian rhythm of labor.

**Methods:**

It was a retrospective propensity score matched cohort study. Parturients were recruited, who delivered term singletons in cephalic position, from seven hospitals in Harvard University Partners Healthcare Systems, 2016–2018. The parturients were divided into two groups, neuraxial analgesia delivery and spontaneous vaginal delivery, the stratification was performed according to labor induction, oxytocin, operative delivery. The parturients in each group were divided into 12 periods in every 2 h based on the birth time of babies. Cosine function fitting was used to verify whether the birth time had the characteristic of circadian rhythm.

**Results:**

In spontaneous vaginal deliveries, the peak of birth time was at 2:00–4:00, and the nadir was at 14:00–16:00, this showed a circadian rhythm presented by a cosine curve fitting with the formula (y = 0.0847 + 0.01711 × cos(− 0.2138 × x + 0.4471). The labor rhythm of NAD (Neuraxial Analgesia Delivery) group changed completely, inconsistent with the cosine curve fitting of the circadian rhythm. The intervention of induction and oxytocin blurred the circadian rhythm of SVD (Spontaneous Vaginal Delivery) group and increased the amplitude of the fluctuation in NAD (Neuraxial Analgesia Delivery) group. The intervention of operative delivery had changed the distribution curve completely both in the SVD (Spontaneous Vaginal Delivery) group and the NAD (Neuraxial Analgesia Delivery) group.

**Conclusions:**

Neuraxial analgesia did affect on circadian rhythm of labor, changed the cosine rhythm of labor with spontaneous vaginal delivery, and this trend was aggravated by the use of induction, oxytocin and operative delivery.

## Background

The coordinated biological behavior in a 24-h time-dependent manner is called circadian clock. Circadian rhythms are the results of the self-regulating biological clocks [1]. If the rhythms are disrupted, they may induce discomfort or dysfunction of the body, and even occurrence of diseases. The chronomedicine was to clarify the rhythm mechanism of physiology and behavior in living organisms, to clarify that adapting to natural rhythms actively may help maintaining health, reducing or avoiding the infestation of pathogenic factors [2]. The labor is a normal physiological process of human beings with its own circadian rhythm. But the results were inconsistent on whether there is rhythm of labor [3–7]. Varea et al. [6] showed the circadian rhythm of labor without intervention as that more births would be given in the morning, based on the inpatient data of Casa de Maternidad in Madrid from 1887 to 1892. Rosaria Cappadona’s report showed no significant impact on the number of deliveries by daytime or night [4]. The night shift, with unconventional light exposure for a long time, also have affected on conception and neonatal health [7]. Labor analgesia is a medical intervention to ease the labor pain. It can relieve the pain, the psychological fear, and the extra oxygen consumption during the process of labor. It can be more beneficial to maternal and neonatal health, and be more and more popular worldwide. However, the labor analgesia has some effects on uterine contraction, labor duration, and some other outcomes of delivery [8]. In fact, we found more baby birth at night than in the daytime. We assumed that childbirth has a circadian rhythm. Labor analgesia has affect on uterine contraction, it may change delivery time, and may affect circadian rhythm of childbirth. The change of circadian rhythm is one of the causes of many diseases. We want to know that whether labor analgesia changes the circadian rhythm of labor, when the use of labor analgesia is so high today. The objective of this study aimed to confirm whether there was delivery rhythm, and to investigate whether the neuraxial labor analgesia or other intervention factors have effects on it.

## Methods

### Data collection

**Fig. 1:**
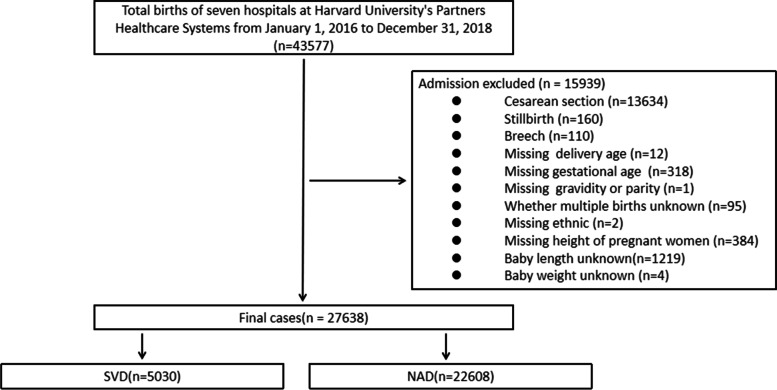
The flow chart of cases enrollment. SVD: Spontaneous Vaginal Delivery; NAD: Neuroaxial Analgesia Delivery

This study retrospectively collected 43,577 delivery data from January 1, 2016 to December 31, 2018 from seven hospitals in Harvard University Partners Healthcare Systems (PARTNERS), including Brigham and Women’s Hospital, Massachusetts General Hospital, Newton-Wellesley Hospital, North Shore Medical Center, Martha’s Vineyard Hospital, Cooly Dickinson Hospital and Nantucket Cottage Hospital in Boston Massachusetts USA. This study was approved by the Partners Human Research Committee with the ethical approval number: 2018P002646. We informed consent from all subjects. All methods were performed in accordance with the relevant regulations. The inclusion criteria covered delivery over 20 weeks, vaginal delivery by cephalic presentation of fetus, the exclusion criteria covered stillbirth, non-cephalic presentation and incomplete data. Total 27,638 vaginal deliveries were included. The flow chart of cases enrollment was shown in Fig. 1.

### Definition of variables

According to the use or non-use of Neuraxial Labor Analgesia (NLA) during labor, the parturients were divided into two groups, Neuraxial Analgesia Delivery (NAD) and Spontaneous Vaginal Delivery (SVD). NAD was defined as a delivery with NLA, such as epidural anesthesia, spinal anesthesia, or combined spinal and epidural anesthesia. SVD was defined as a delivery without NLA. The number of gravidity included this current pregnancy, and the number of parity excluded this current delivery. Maternal BMI (Body Mass Index) referred to the weight (kg) / height (m)^2^ of a parturient before labor. Preterm delivery referred to the delivery at 20–36^+ 6^ gestational weeks. Labor induction referred to the use of medications or other methods to stimulate uterine contractions to have a vaginal delivery. Oxytocin was the stimulating medicine for uterine contractions during labor. Operative delivery referred to the delivery with forceps or vacuum extractor. The stratification was performed according to labor induction, oxytocin, operative delivery. The parturients in each group were divided into 12 periods in every 2 h based on the birth time of babies: 0:00–2:00, 2:00–4:00, 4:00–6:00, 6:00–8:00, 8:00–10:00, 10:00–12:00, 12:00–14:00, 14:00–16:00, 16:00–18:00, 18:00–20:00, 20:00–22:00, 22:00–0:00.

### Statistical analysis

The continuous data was tested for normality using the Kolmogorov-Smirnov test. The data description was presented as mean ± standard deviation (Mean ± SD) or median (interquartile ranges (IQR)) for continuous variables and percentages for categorical variables. Group difference was analyzed using One-way analysis of variance (ANOVA) for normally distributed variables and Kruskal-Wallis H test for non-normally distributed variables. Chi-square test was used for categorical variables as group comparison. Case-control matching was performed by propensity score, which uses logistic regression analysis to match based on multiple parameters. The data of SVD group was used to match the data of the NAD group. The SVD group was matched 1-to-1 following the near neighbor rule with a caliper width equal to 0.00001 of the logit of the standard deviation of the propensity score. After matching, the balance between the SVD group and the NAD group was assessed for each variable by examining the standardized differences. A standardized difference < 0. 05 was considered to a negligible imbalance between the two groups. After matching, the number of women in SVD group and NAD group at different birth time was compared, and the subgroup analysis was conducted according to whether induction of labor, oxytocin and operative delivery were applicable. All statistical tests of hypotheses will be two sided and the criterion for statistical significance is α = 0.05. Statistical analyses were done with SPSS version 17.0 software (IBM).

The cosine function fitting was used to verify whether the data had the characteristic of rhythm. The cosine function equation for fitting is *F*(t) = *M* + *A* cos(*ω* + *φ*), where *M* is the median value; *A* is the amplitude of the rhythmic oscillation; *φ* is the peak phase, which is the angle converted from the moment when the oscillation reaches the peak. According to the *ω* angle (360°/24 h), the frequency analysis can be converted to ZT time (24 h). This study used cosine function fitting to verify whether the labor time had the characteristic of circadian rhythm. The rhythmic parameters were from the MATLAB software.

## Results

According to the collected data, there were 29,943 vaginal deliveries and 13,634 cesarean deliveries in PARTNERS from 2016 to 2018. The rate of cesarean delivery was 31.3%. After excluding the deliveries that met the exclusion criteria, there were total 27,638 vaginal deliveries left. Among these 27,638 vaginal deliveries, 22,608 parturients delivered with NLA (the NAD group), which accounted for 81.8%, and 5030 parturients delivered spontaneously without NLA (the SVD group), which accounted for 18.2%. The rate of parturients with labor induction in NAD group was 33.0% (*n* = 7536), higher than 16.1% (*n* = 810) of the SVD group (*P* < 0.001). The rate of parturients with oxytocin in the NAD group was 55.3% (*n* = 12,513), higher than 18.9% (*n* = 949) of the SVD group (*P* < 0.001). The rate of operative deliveries in the NAD group was 7.7% (*n* = 1744), higher than 1.9% (*n* = 95) of the SVD group (*P* < 0.001). The demographic and obstetric difference between the two groups was statistically significant in terms of maternal age, gravidity, parity, maternal height, maternal BMI, baby height, multiple pregnancy, induction, oxytocin, operative delivery, ethnics (Table 1). After use of Propensity Score Matching (PSM), we found that 3054 parturients of SVD group matched to 3054 parturients of NAD group, and the baseline characteristics of demographics and obstetrics between the two groups were similar for most variables, except for maternal BMI and ethnics (Table 2). We compared the SVD group with the NAD group in term of circadian rhythm after use of Propensity Score Matching.

**Table 1:** Baseline characteristics of participations

	SVD *n* = 5030	NAD *n* = 22,608	*χ*^*2*^*/Z*	*P value*
Age ^a^	32.00 (28.00,35.00)	32.00 (29.00,35.00)	−2.889	0.004
Gestational age (weeks)^a^	39.57 (38.71,40.29)	39.57 (38.71,40.29)	0.614	0.539
Gravidity^a^	2.00 (2.00,4.00)	2.00 (1.00,3.00)	15.750	< 0.001
Parity^a^	1.00 (0.00,2.00)	1.00 (0.00,1.00)	25.976	< 0.001
Maternal height (cm) ^a^	162.60 (157.50,167.60)	163.00 (160.00,168.00)	−5.915	< 0.001
Maternal BMI (kg/(m)^2^)^a^	28.95 (26.08,32.53)	29.21 (26.42,32.89)	−3.422	0.001
Baby height (cm) ^a^	50.00 (48.30,52.00)	50.50 (48.30,52.10)	−5.013	< 0.001
Baby weight (kg) ^a^	3.35 (3.04,3.65)	3.35 (3.04,3.66)	−0.160	0.873
Preterm delivery^b^	287 (5.7%)	1261 (5.6%)	0.128	0.721
Multiple pregnancy^b^	29 (0.6%)	490 (2.2%)	56.513	< 0.001
Induction^b^	810 (16.1%)	7536 (33.0%)	579.495	< 0.001
Oxytocin^b^	949 (18.9%)	12,513 (55.3%)	2191.814	< 0.001
Operative delivery^b^	95 (1.9%)	1744 (7.7%)	224.805	< 0.001
Ethnic^b^			195.759	< 0.001
White	2820 (56.1%)	14,968 (66.2%)		
Black	597 (11.9%)	1818 (8.0%)		
others	1613 (32.1%)	5822 (25.8%)		

Data presented as n (%) or median (interquartile range); *SVD* Spontaneous Vaginal Delivery; *NAD* Neuroaxial Analgesia Delivery

^a^The non-normal distribution continuous data were presented as median (interquartile range), the data were compared using Mann-Whitney U test (Z)

^b^The categorical data were presented as numbers (frequencies, %), Chi-square test was used for categorical data (χ^2^)

**Table 2:** Baseline characteristics of participations after the propensity score matched

	SVD *n* = 3054	NAD *n* = 3054	χ2/Z	*P* value
Age^a^	32.00 (29.00,35.00)	32.00 (29.00,35.00)	−0.279	0.781
Gestational age (weeks)^a^	39.57 (38.86,40.29)	39.57 (38.86,40.29)	−0.032	0.975
Gravidity^a^	2.00 (2.00,3.00)	2.00 (2.00,3.00)	−1.477	0.140
Parity^a^	1.00 (0.00,1.00)	1.00 (0.00,1.00)	1.171	0.242
Maternal height (cm) ^a^	163.00 (160.00,167.60)	163.80 (160.00,167.60)	−0.965	0.335
Maternal BMI (kg/(m)^2^)^a^	28.47 (25.77,32.01)	28.56 (26.10,32.13)	−4.317	< 0.001
Baby height (cm) ^a^	50.20 (48.30,52.00)	50.50 (48.30,52.10)	−0.989	0.323
KBaby weight (kg) ^a^	3.37 (3.07,3.65)	3.35 (3.08,3.64)	−0.071	0.943
Preterm delivery^b^	130 (4.3%)	103 (3.4%)	3.253	0.071
Multiple pregnancy^b^	12 (0.4%)	11 (0.4%)	0.044	0.835
Induction^b^	469 (15.4%)	431 (14.1%)	1.882	0.170
Oxytocin^b^	626 (20.5%)	597 (19.5%)	0.860	0.354
Operative delivery^b^	49 (1.6%)	58 (1.9%)	0.771	0.380
Ethnic^b^			7.212	0.027
White	1984 (65.0%)	2083 (68.2%)		
Black	229 (7.5%)	208 (6.8%)		
others	841 (27.5%)	763 (25.0%)		

Data was presented as n (%) or median (interquartile range); *SVD* Spontaneous Vaginal Delivery, *NAD* Neuroaxial Analgesia Delivery

^a^The non-normal distribution of continuous data were presented as median (interquartile range), the data were compared using Mann-Whitney U test (Z)

^b^The categorical data were presented as numbers (frequencies, %), Chi-square test was used for categorical data (χ^2^)

**Fig. 2:**
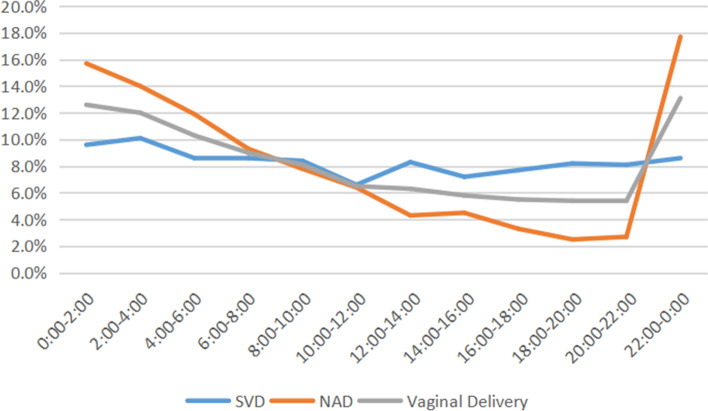
Percentage of vaginal delivery in each time point. NAD, Neuroaxial Analgesia Delivery; SVD, Spontaneous Vaginal Delivery. n (SVD) = 3054; n (NAD) = 3054

The distribution of the birth time of SVD group without NLA showed a diurnal variation pattern, with a peak at 2:00–4:00 in the early morning, which accounted for 10.1% of births of SVD group, and with a nadir at 10:00–12:00, which accounted for 6.6% of births of the group. There were more babies born at night (20:00–8:00, 53.6%) than at daytime (8:00–20:00, 46.4%). The distribution of birth time of NAD group with NLA showed a peak at 22:00–0:00, which accounted for 17.7%, and a nadir at 18:00–20:00, which accounted for 2.5%. The peak of the birth time of SVD group (2:00–4:00 in the early morning) was later than that of NAD group (22:00–0:00), while the nadir of the birth time of SVD group (10:00–12:00) was earlier than that of NAD group (18:00–20:00). Both the time period and the proportion of the peak and nadir of the two groups were obviously different from each other. There were more babies from NAD group born at night (20:00–8:00, 71.3%) than at daytime (8:00–20:00, 28.7%), the difference between the two groups was statistically significant (*P* < 0.001). The birth time of NAD group changed completely compared with the birth time of SVD group. The proportion of births in SVD group was significantly higher than that in NAD group at 12:00–14:00, 14:00–16:00, 16:00–18:00, 18:00–20:00, 20:00–22:00 (*P* < 0.001). The proportion of births in SVD group at 22:00–0:00, 0:00–2:00, 2:00–4:00, 4:00–6:00 was significantly lower than that in NAD group (*P* < 0.001). The distribution of birth time of the SVD group and NAD group was showed in Table 3 and Fig. 2.

**Table 3:** Distribution of Birth Time of Parturients with Vaginal Delivery

Birth Time	SVD n (%)	NAD n (%)	Vaginal Delivery n (%)	χ2	*P* value
0:00–2:00	292 (9.6%)	479 (15.7%)	771 (12.6%)	51.908	<0.001
2:00–4:00	307 (10.1%)	428 (14.0%)	735 (12.0%)	22.645	<0.001
4:00–6:00	264 (8.6%)	364 (11.9%)	628 (10.3%)	17.748	<0.001
6:00–8:00	264 (8.6%)	283 (9.3%)	547 (9.0%)	0.725	0.395
8:00–10:00	257 (8.4%)	239 (7.8%)	496 (8.1%)	0.711	0.399
10:00–12:00	203 (6.6%)	194 (6.4%)	397 (6.5%)	0.218	0.640
12:00–14:00	254 (8.3%)	131 (4.3%)	385 (6.3%)	41.940	<0.001
14:00–16:00	219 (7.2%)	136 (4.5%)	355 (5.8%)	20.603	<0.001
16:00–18:00	234 (7.7%)	101 (3.3%)	335 (5.5%)	55.867	<0.001
18:00–20:00	251 (8.2%)	76 (2.5%)	327 (5.4%)	98.952	<0.001
20:00–22:00	246 (8.1%)	83 (2.7%)	329 (5.4%)	85.354	<0.001
22:00–0:00	263 (8.6%)	540 (17.7%)	803 (13.1%)	110.016	<0.001

*SVD* Spontaneous Vaginal Delivery *NAD* Neuroaxial Analgesia Delivery. McNemar’s test was used

**Fig. 3:**
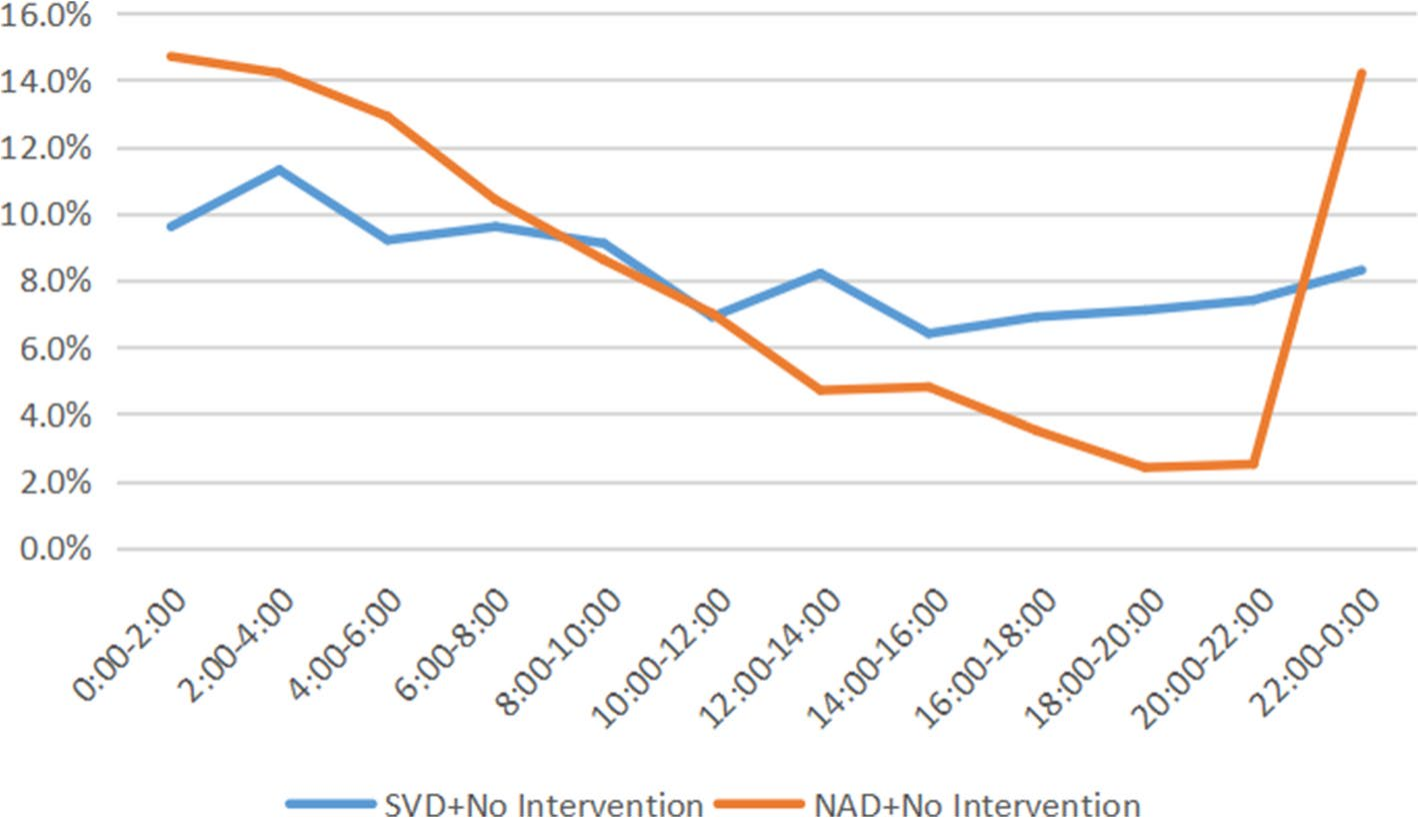
Distribution of birth time in women without intervention. SVD, Spontaneous Vaginal Delivery; NAD, Neuroaxial Analgesia Delivery. n (SVD) = 2229; n (NAD) = 2221

**Fig. 4:**
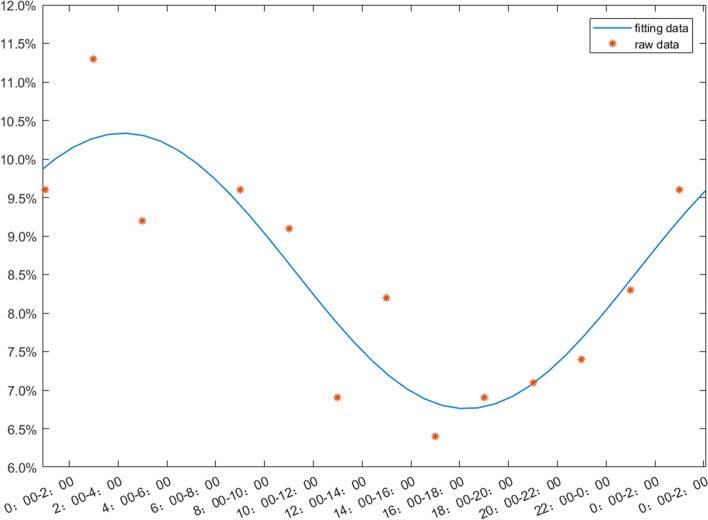
The raw data and the cosine function fitting diagram of the vaginal delivery group without intervention, y = 0.0847 + 0.01711 × cos(− 0.2138 × x + 0.4471). *n* = 2229

There were 2221 parturients in NAD group who delivered without use of induction, oxytocin and operative delivery. We found that the number of births increased sharply from 20:00 to 2:00, and the number of births gradually declined after that period of time (Fig. 3). There were 2229 parturients who delivered without any interventional factors, the peak of birth time was at 2:00–4:00, and the nadir was at 14:00–16:00, this showed a circadian rhythm presented by a cosine curve fitting, with the formula as (y = 0.0847 + 0.01711 × *cos(− 0.2138 × x + 0.4471)* (Fig. 4). The labor rhythm of NAD group changed completely, inconsistent with the cosine curve fitting of the circadian rhythm.

The SVD group and the NAD group were stratified based on the three medical interventional factors, including induction, oxytocin and operative delivery. The distribution of birth time of SVD subgroup with use of induction showed the peak was at 22:00–0:00(10.9%), and the nadir was at 10:00–12:00(4.9%), the peak and the nadir were changed, compared with SVD subgroup without induction. The peak of birth time in NAD subgroup with use of induction was at 22:00–0:00(26.0%), and the nadir was at 18:00–20:00(2.8%). The intervention of induction blurred the circadian rhythm of SVD group and increased the amplitude of the fluctuation in NAD group.

The distribution of birth time of SVD group with use of oxytocin showed the peak at 18:00–20:00(11.8%), and the nadir was at 6:00–8:00(5.6%), the peak and the nadir were changed, compared with SVD subgroup without oxytocin. It could be seen that the peak of birth time in NAD subgroup with use of oxytocin was at 22:00–0:00(30.7%), and the nadir was at 20:00–22:00(1.3%). The intervention of oxytocin also blurred the circadian rhythm of SVD group, and increased the amplitude of the fluctuation of NAD group, similar in induction intervention. The distribution of birth time of SVD subgroup with induction and oxytocin had changed, the peak value of NAD subgroup with interventional factors was higher, and the nadir value of NAD subgroup was lower than that of subgroup without induction and oxytocin, which showed sharper fluctuations.

**Fig. 5:**
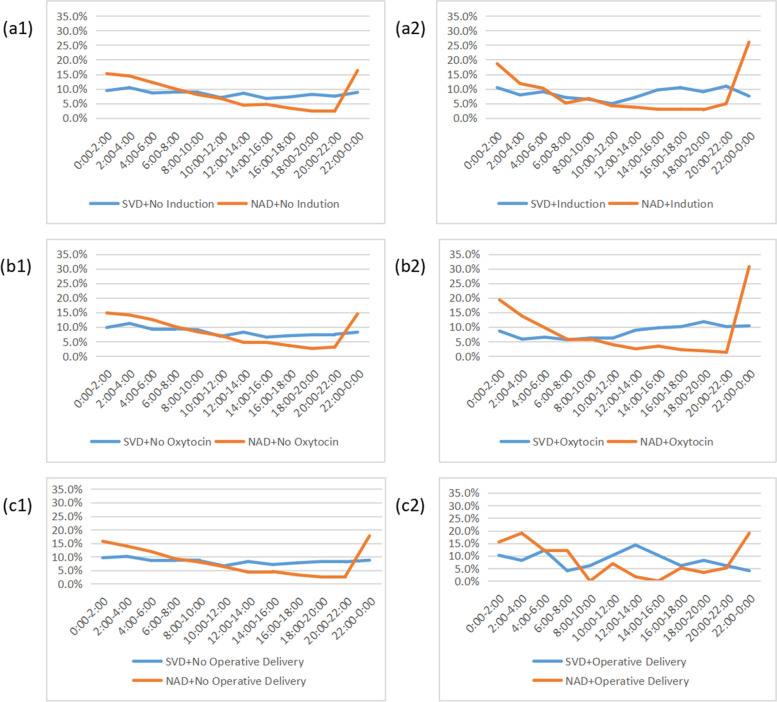
The factors affecting circadian rhythm of vaginal delivery. SVD, Spontaneous Vaginal Delivery; NAD, Neuroaxial Analgesia Delivery. a1. Distribution of birth time in parturients without induction, n (SVD) = 2585; n (NAD) = 2623; a2. Distribution of birth time in parturients with induction, n (SVD) = 469; n (NAD) = 431; b1. Distribution of birth time in parturients without oxytocin, n (SVD) = 2428; n (NAD) = 2457; b2. Distribution of birth time in parturients with oxytocin, n (SVD) = 626; n (NAD) = 597; c1. Distribution of birth time in parturients without assisted operation, n (SVD) = 3005; n (NAD) = 2996; c2. Distribution of birth time in parturients with assisted operation, n (SVD) = 49; n (NAD) = 58

The distribution of birth time of SVD subgroup with operative delivery showed the peak at 12:00–14:00(14.3%), and the nadir at 6:00–8:00, 22:00–0:00(4.1%). It could be seen that the peak of birth time in NAD group with operative delivery was at 22:00–0:00, 2:00–4:00(19.0%), and the nadir was at 8:00–10:00, 14:00–16:00(0.0%), the peak and the nadir were changed completely in both the SVD group and the NAD group. The intervention of operative delivery had changed circadian rhythm completely (Fig. 5).

## Discussion

The biological rhythm referred to the phenomenon that the physiology and behavior change periodically in all living things. It was the inherent feature of life activities, even in the absence of light, but can be interfered by the external environment [9]. The circadian rhythm system aligned biological activities with environmental patterns to optimize function and health, more than 300 life activities of mammals and human beings, such as sleep, body temperature, heart rate, blood pressure, preterm delivery, hormone secretion, all showed rhythmic fluctuations in 24 h [10–13]. Labor also has a circadian rhythm. The circadian rhythm of labor was altered due to the intervention of labor induction, oxytocin and operative delivery.

In order to find out whether a life process has its own circadian rhythm, it was necessary to divide the 24 h into several time periods, to detect the fluctuation value of each time period. If the diurnal fluctuations of these time periods were in accordance with cosine function, then it could be determined that it has circadian rhythm [14]. In this study, the 24 h was divided into 12 periods. Our study showed an obvious cosine circadian rhythm in spontaneous vaginal delivery, the proportion of childbirth reached the peak at 2:00–4:00, and the nadir at 10:00–12:00. They had a higher proportion of births in early morning, and this was consistent with some other studies. Varea, C [6] showed the peak period of childbirth was at 4:00 at the end of the nineteenth century in Madrid, Spain. Charles, E [15] showed the the peak was at 0:00–4:00 am from 1949 to 1951 in Birmingham, England. Because there were no other medical interventions and less influence from the surrounding environment such as artificial lighting at that time, so it was closer to the natural state. The earlier peak and nadir of the birth time may be related to modern rest-activity rhythm and illumination of modern society, which was different from that of a hundred years ago when there was no modern obstetric intervention and artificial lighting [6]. The illumination at night would interfere with sleep and lead to the change of circadian rhythm [16]. The secretion of melatonin could be inhibited in a few minutes by light reversibly [17]. Humans were more sensitive to light than they thought of themselves, and the difference in individual sensitivity to night light may disrupt circadian rhythm [2]. Perhaps these were related to the postponing of the peak of childbirth.

In our study, the proportion of births was 53.6% between 20:00 and 8:00, and 46.4% between 8:00 and 20:00. There were more babies delivered in the early morning, less babies delivered in the afternoon if no interventions were made. The hypothalamic-pituitary-adrenal cortex axis (HPA axis) released cortisol in a circadian rhythm pattern [18]. Such a rhythmic releasing process also showed in the parturients. Cortisol induced the production of chemokines and cytokines in the myometrium during pregnancy, followed by a cascade of intrauterine inflammation that were triggered [19]. The proportion of births became lower with the decreased secretion of cortisol, on the contrary, the proportion of births became higher with the increased secretion of cortisol. The melatonin was also related to the circadian rhythm. Its secretion increased at night and reached a peak at 1:00–4:00, and gradually decreased during the daytime [20], which was synchronized with the circadian rhythm of labor. Myometrial cells had melatonin receptors that bound to oxytocin and norepinephrine, which caused the contraction of uterine muscles. The uterine contraction was more intense with the in creased secretion of melatonin at night [21, 22], and this increased the proportion of births in the early morning.

Neuraxial analgesia, including spinal, epidural and combined spinal-epidural (CSE) techniques, was a widely used method of sensory block anesthesia, and was used to relieve the pain of labor effectively. It was the most popular method and the most effective method for labor analgesia, and 81.8% of vaginal deliveries were using it in our study. Although there are still some doubts that neuraxial analgesia may prolong labor progression and thus affect pelvic floor muscle function [23], current studies show that neuraxial analgesia, especially epidural anesthesia, does not increase the rate of postpartum urinary incontinence [24]. Therefore, it also reduces maternal concerns about the technology and increases the utilization rate of neuraxial analgesia. The increased use of NLA was accompanied by other medical interventions, such as induction, oxytocin, operation delivery, and it was not associated with increased adverse outcomes [25]. The proportion of births at 20:00–8:00 increased from 53.6 to 71.3% through pharmacological intervention of neuraxial analgesia. The labor rhythm of the NAD group with NLA changed completely, as it was not in the pattern of cosine circadian rhythm, the peak of birth time changed, the proportion of births at the peak period increased from 10.1 to 17.7%, while the nadir decreased from 6.6 to 2.5%. The anesthetic agents had a destructive effect on the circadian rhythm of mammals, the circadian clock was slowed down and inhibited by the anesthetic agents, and the normal circle of circadian rhythm [26]. Studies indicated that there were changes in melatonin when anesthetic agents were used [27]. Melatonin was regulated by suprachiasmatic nuclei (SCN) and the adrenergic pathway [28–30]. The anesthetic agents had an inhibitory effect on the secretion from SCN to the paraventricular nucleus, it also affected sleep homeostasis and rest-activity rhythms [31]. The change of circadian rhythm after the use of NLA, might be related to the effect of anesthetic agents on SCN, paraventricular nucleus, melatonin, adrenergic pathway, photoperiod, rest-activity rhythm and uterine contractions.

Labor induction, oxytocin and operative delivery also had a great impact on the circadian rhythm of labor. Labor analgesia was used when cervical dilation was about 3 cm in our study whether daytime or midnight. The use of oxytocin was not fixed for daytime or midnight, and we use it when there are indications for oxytocin use, such as weak contractions. But induction was usually carry out in the morning. In this study, the effect of induced labor on circadian rhythm was less than that of NLA. Medical interventions had an impact on the labor rhythm, because it was also related to the working shift of the medical staff, and the schedule of the parturients. Not only doctors, but also parturients preferred to induction of labor in the morning, even the parturients who were admitted to the hospital at night. They did not like the sleep deprivations associated with the labor induction. It would increase the rate of vaginal delivery when the prostaglandin was used in the morning [32].

The effect of NLA on the circadian rhythm of labor was magnified by the use of induction, the peak of birth time increased from 16.3 to 26.0%, the amplitude of rhythmic fluctuation increased, and the peak point of rhythm was concentrated in 22:00–2:00, indicating that the use of NLA and induction led to concentrated labor in the midnight, earlier than the peak point of 2:00–4:00 in spontaneous vaginal delivery without any intervention. The effect of intervention with the use of oxytocin on labor rhythm was similar and greater to that of intervention with the use of induction. The rhythm of labor was completely disrupted by assisted operative intervention, not only in NAD group, but also in SVD group. It may be related to the artificial reduction of the second stage of labor by forceps or vacuum. Whatever intervention it was, induction, oxytocin or operative delivery, its effects on the circadian rhythm of SVD group were greater than that of the NAD group, and the cosine curve changed significantly.

The strength of the study was that the human labor circadian rhythm is consistent with cosine curve. Neuraxial labor analgesia did affect on it, changed the cosine rhythm of spontaneous vaginal delivery, and this trend was aggravated by the use of induction, oxytocin, and operative delivery. The limitation of this study was that we did not consider the effect of time, duration and intensity of medical intervention on the circadian rhythm. It was still unclear how NLA affected the circadian rhythm of labor, and how other factors that had effect on the circadian rhythm interacted with each other. Prospective clinical studies and basic studies are needed to be done so as to understand the effect of interventional factors on the circadian rhythm of labor, to promote spontaneous delivery rate, and to reduce the occurrence of dystocia as well as the maternal and perinatal adverse outcomes.

## Data Availability

The datasets used in the current study are available from the corresponding author on reasonable request.

## Abbreviations

NLA: Neuraxial Labor Analgesia
NAD: Neuraxial Analgesia Delivery
SVD: Spontaneous Vaginal Delivery
PSM: Propensity Score Matching
SCN: Suprachiasmatic Nuclei
